# Plasma Levels of Keratinocyte Growth Factor Are Significantly Elevated for 5 Weeks After Minimally Invasive Colorectal Resection Which May Promote Cancer Recurrence and Metastasis

**DOI:** 10.3389/fsurg.2021.745875

**Published:** 2021-11-08

**Authors:** H. M. C. Shantha Kumara, Abhinit Shah, Hiromichi Miyagaki, Xiaohong Yan, Vesna Cekic, Yanni Hedjar, Richard L. Whelan

**Affiliations:** ^1^Division of Colon and Rectal Surgery, Department of Surgery, Lenox Hill Hospital, Northwell Health, New York, NY, United States; ^2^Gastroenterological Surgery, Osaka University, Suita, Japan; ^3^Donald and Barbara Zucker School of Medicine at Hofstra/Northwell, Hempstead, NY, United States

**Keywords:** minimally invasive colorectal resection, KGF, plasma levels, metastasis, angiogenesis

## Abstract

**Background:** Human Keratinocyte Growth Factor (KGF) is an FGF family protein produced by mesenchymal cells. KGF promotes epithelial cell proliferation, plays a role in wound healing and may also support tumor growth. It is expressed by some colorectal cancers (CRC). Surgery's impact on KGF levels is unknown. This study's purpose was to assess plasma KGF levels before and after minimally invasive colorectal resection (MICR) for CRC.

**Aim:** To determine plasma KGF levels before and after minimally invasive colorectal resection surgery for cancer pathology.

**Method:** CRC MICR patients (pts) in an IRB approved data/plasma bank were studied. Pre-operative (pre-op) and post-operative (post-op) plasma samples were taken/stored. Late samples were bundled into 7 day blocks and considered as single time points. KGF levels (pg/ml) were measured *via* ELISA (mean ± SD). The Wilcoxon paired *t*-test was used for statistical analysis.

**Results:** Eighty MICR CRC patients (colon 61%; rectal 39%; mean age 65.8 ± 13.3) were studied. The mean incision length was 8.37 ± 3.9 and mean LOS 6.5 ± 2.6 days. The cancer stage breakdown was; I (23), II (26), III (27), and IV (4). The median pre-op KGF level was 17.1 (95 %CI: 14.6–19.4; *n* = 80); significantly elevated (*p* < 0.05) median levels (pg/ml) were noted on post-op day (POD) 1 (23.4 pg/ml; 95% CI: 21.4–25.9; *n* = 80), POD 3 (22.5 pg/ml; 95% CI: 20.7–25.9; *n* = 76), POD 7–13 (21.8 pg/ml; 95% CI: 17.7–25.4; *n* = 50), POD 14–20 (20.1 pg/ml; 95% CI: 17.1–23.9; *n* = 33), POD 21–27 (19.6 pg/ml; 95% CI: 15.2–24.9; *n* = 15) and on POD 28–34 (16.7 pg/ml; 95% CI: 14.0–25.8; *n* = 12).

**Conclusion:** Plasma KGF levels were significantly elevated for 5 weeks after MICR for CRC. The etiology of these changes is unclear, surgical trauma related acute inflammatory response and wound healing process may play a role. These changes, may stimulate angiogenesis in residual tumor deposits after surgery.

## Introduction

Surgery remains the mainstay of treatment for colon and rectal cancer (CRC). Unfortunately, 30–40% of patients who undergo “curative” resection harbor micrometastases after surgery that lead to tumor recurrences. Neoadjuvant and adjuvant chemotherapy, radiotherapy and immunotherapy have improved survival, however, despite these treatments a good proportion of patients eventually succumb (1–3). Interestingly, surgery, necessary to remove the primary tumor, may transiently create an environment, *via* immunosuppression or other surgery related alterations, that is conducive to the growth of residual cancer microfoci or circulating viable tumor cells (4–6). There is clinical and murine evidence of accelerated tumor growth early after surgery that supports this hypothesis (4, 7–9). If a tumor supportive milieu exists after surgery then it is logical to develop anti-cancer treatments for use during the first 4–6 weeks after surgery which is a time period not currently being utilized (10, 11). One proposed mechanism for accelerated tumor development after surgery are long duration proangiogenic and tumor stimulatory plasma protein changes that may stimulate tumor growth in residual tumor microfoci.

The vast majority of plasma protein changes after surgery last hours or days (IL-1, TNF, IL-6, CRP, etc.) (12–14), however, in the last decade, increases in blood levels of at least 12 progangiogenic proteins have been noted to persist for 3 to 5 weeks after resection of colorectal cancer (CRC) (15–25). *In vitro* studies utilizing endothelial cells (EC's), critical to angiogenesis, suggest the net effect of plasma compositional changes is proangiogenic. Plasma from the 2nd and 3rd weeks after minimally invasive colorectal cancer resection (MICR) stimulates endothelial cell (EC) proliferation, invasion and migration which are required for neovascularization (15, 26). The principal source of the added protein in the blood is thought to be the healing surgical wounds (27). Efforts to further characterize the surgery-related plasma protein changes continue. Keratinocyte Growth Factor (KGF) is a good candidate for study because it is involved with wound healing and also impacts epithelial cell growth.

KGF, also known as FGF-7, is a member of the Fibroblast Growth Factor family. It is involved in a variety of biological processes, including cell growth, morphogenesis and tissue repair. It is generated by fibroblasts and other mesenchymal cells and works exclusively through the FGFR2b and FGFR2c receptors that are expressed mainly by epithelial cells (28, 29). KGF's effects are largely paracrine since it effects epithelium and, therefore, it is a mediator of mesenchymal-epithelial interactions (30, 31). KGF expression is stimulated by IL1 and IL6 (28, 29, 32) as well as by platelet-derived growth factor BB (PDGF BB) and transforming growth factor α (TGFα). KGF is induced in the setting of both acute and chronic injury and there is also strong evidence that KGF plays an role in wound healing (33, 34).

The binding of fibroblast derived KGF to KGFR on epithelial cells promotes re-epithelialization after injury. Exogenous KGF facilitates both skin wound and anastomotic healing in rodents and mice (35–37). Wound healing is impaired in KGF knockout mice and after KGF blockade (36). There is also evidence that KGF impacts wound angiogenesis (36, 38, 39). Diminished angiogenesis and lower VEGF levels been noted in wounds of KGF knockout mice (vs. wild type mice) (36). Given its role in epithelial wound healing it is not surprising that KGF impacts cancer growth as well.

KGF has been shown to facilitate the growth of KGFR expressing epithelial cancers. Stomach, colon, pancreas, ovarian, and other cancers have been shown to express this receptor (40–44). Although the peri-tumor stroma is the principal source of KGF, some tumor cells also express this protein (42, 45–47). KGF has been shown to be a tumor cell mitogen and to promote cancer cell motility, VEGF production, and tumor angiogenesis (41, 43, 47, 48).

Given KGF's role in cancer development and the hypothesis that proteins with long duration plasma elevations after surgery originate in the wound, it is logical to perform an in depth perioperative plasma study for KGF in patients with CRC. Surgery's impact on KGF levels is not well-documented. The purpose of this study was to determine pre-operative (pre-op) and post-operative plasma KGF levels for 5 weeks after minimally invasive resection (MICR) of CRC.

## Methods

### Study Population

Colorectal cancer (CRC) patients who underwent elective minimally invasive colorectal resection (MICR) at Mount Sinai West Hospital and New York Presbyterian Hospital between 2006 and 2013 who had been enrolled in an IRB approved multicenter prospective tissue and data bank (Institutional Review Board of the Mount Sinai School of Medicine, New York; IRB Reference No: GCO1: 16-2619 and Institutional Review Board of the Columbia University Medical Center, New York; IRB Reference No: AAAA4473) were eligible for the present study. The generally stated purpose of the tissue and data bank was to study the physiologic, immunologic, and oncologic ramifications of major abdominal surgery. Enrolled patients underwent minimally invasive laparoscopic-assisted (LAP) or hand assisted laparoscopic (HAL) surgery; none of the patients received a novel drug or other therapy. The indications and type of surgery as well as the demographic, operative, and short-term recovery data were prospectively collected for all the patients. Intraoperative or recently transfused patients, immunosuppressed patients (medication-related and HIV+), and those who received radio- or chemotherapy within 6 weeks of surgery were excluded. Patients undergoing urgent or emergent surgery were also excluded. Clinical, demographic, and operative data were obtained from office charts as well as operative and pathology records.

### Blood Sampling and Processing

As per the tissue banking protocol, research dedicated blood samples were obtained from consenting CRC MICR patients pre-operatively, on post-operative days (POD) 1, 3, and at different time points beyond the first week following surgery. Only those patients for whom adequate volumes of plasma were available for the pre-op, POD 1, POD 3, and at least 1 late time point were enrolled. Since post-discharge blood samples were taken at the time of follow-up office visits which varied considerably as to the specific post-operative day they occurred, it was necessary to “bundle” the late samples into 7 day time blocks (POD 7–13, POD 14–20, POD 21–27, and POD 28–34). Specimens from these 7 day periods were considered as single time points. The post-operative time blocks were made based on the time of samples collection. Of note, the “*n*” for each of the post-hospital discharge time points is well less than the starting “*n*” of 80 because patients were generally seen only 1 to 2 times during the first 5 weeks following surgery (and not on a weekly basis). Specimens were collected in heparin-containing tubes, and processed within 5–6 h of collection. After centrifugation at 450 × g for 10 min, the plasma was frozen and stored at −80°C until the assays were performed.

### Plasma KGF Analysis

Blood samples were obtained pre-operatively, at POD 1 or POD 3, and at least one late time point (POD 7–34) into heparin-containing blood collection tubes and processed within 5–6 h. The plasma samples were isolated *via* centrifugation (450XG for 10 min at 6°C) and stored in 500 ul aliquots. Plasma KGF levels were analyzed in duplicate using a commercially available enzyme-linked immunosorbent assay (R and D Systems, Minneapolis, MN, USA) according to the manufacturer's instructions. Briefly, 100 μL plasma samples in duplicate were used for the assay. The human KGF standard stock solution (20,000 pg/mL) was made with deionized water by reconstituting ELISA kit accompanied standard. Samples were prepared for analysis following the KGF assay kit (Catalog Number DKG00) protocol provided with the kit. A standard curve for each plate was made by using dilution series of KGF standard stock solution where the 2000 pg/mL standard serves as the high standard. The calibrator diluent serves as the zero standards (0 pg/mL). The optical density of ELISA plate was determined at the end of the reaction using an automated microplate reader (Synergy2; Bio-Tek Instruments, Inc., Winooski, VT, USA) set to 450 nm and plasma KGF concentrations were determined using the standard curve. The standard curve was created using software capable of generating a log/log curve-fit and protein concentrations are reported as pg/ml.

### Statistical Analysis

Demographic and clinical data are expressed as the mean ± SD for continuous variables. In the analysis of pre-op vs. post-op KGF levels in CRC patients, the Wilcoxon signed rank test was used for analysis. To exhibit significant differences between Pre-op vs. post-op values the data is depicted in a bar graph expressing KGF levels as median and 75% quartile range. Since the “*n*” at the later timepoints varies and progressively decreases, a unique pre-op results bar that depicts the median values is included for each timepoint. Comparisons of KGF levels of male vs. female patients and hand-assisted vs. laparoscopic patients were carried out using the Mann Whitney test. Correlation between post-op plasma KGF levels and age, incision size and length of surgery was assessed by the Spearman's rank correlation coefficient (rs). Data analysis was performed using SPSS version 15.0 (SPSS, Inc., Chicago, IL, USA).

## Results

A total of 80 CRC patients (37 males, 43 females; mean age 65.8 ± 13.3 years) who underwent MICR were included in the study. Of the 80 patients, 49 patients (61%) had colon cancer, while 31 patients (39%) had rectal malignancies. Of note, the colon cancer subgroup was significantly older than the rectal cancer cohort (68.2 ± 12.5 vs. 62.1 ± 14.1). The majority of patients underwent laparoscopic-assisted resection (58%), whereas the remainder (42%) underwent a hand-assisted or hybrid laparoscopic procedure. The breakdown of operations performed was as follows: Right colectomy, 34%; LAR/anterior resection, 24%; sigmoid/rectosigmoid, 21%; subtotal/total colectomy, 9%; transverse colectomy, 6%, left colectomy, 4%; and APR, 2% (Table 1). The mean incision length was 5.9 ± 1.9 cm for LAP and 10.97 ± 3.3 cm for HAL, and the mean length of stay was 6.5 ± 2.6 days. The final cancer stage breakdown was as follows: Stage I (23); Stage II (27); Stage III (26); and Stage IV (4). There were no perioperative deaths and there were no superficial, deep, or organ space surgical site infections noted. Eleven post-operative complications were noted and included: ileus (5), urinary tract infection (3), C-Diff colitis (1), phlebitis (1), and seroma (1).

**Table 1:** Demographic and clinical characteristics of the study population.

**Age, years (mean ± SD)**	**65.8 ± 13.3**
**Sex (*n*):**	
**Male**	**37 (46.0%)**
**Female**	**43 (54.0%)**
**Incision length (entire patient population), cm (mean ± SD)**	**8.37 ± 3.9**
**Incision length (lap procedure group), cm (mean ± SD)**	**5.91 ± 1.9**
**Incision length (hand procedure group), cm (mean ± SD)**	**10.97 ± 3.3**
**Operative time, min (mean ± SD)**	**295.0 ± 129.9**
**Length of stay, days (mean ± SD)**	**6.5 ± 2.6**
**Pathological stage (*n*)**	
**Stage I**	**23 (29%)**
**Stage II**	**27 (34%)**
**Stage III**	**26 (32%)**
**Stage IV**	**4 (5%)**
**Type of resection (*n*):**	
**Right**	**27 (34.0%)**
**LAR/AR (16/3)**	**19 (24.0%)**
**Sigmoid /recto-sigmoid (14/3)**	**17 (21.0%)**
**Total/sub total (3/3)**	**6 (9.0%)**
**Transverse**	**5 (6.0%)**
**Left**	**3 (4.0%)**
**APR**	**2 (2.0%)**
**Surgical method:**	
**Laparoscopic-assisted (LAP)**	**46 (58.0%)**
**Hand-assisted/hybrid laparoscopic (HAL)**	**34 (42.0%)**

**Figure 1:**
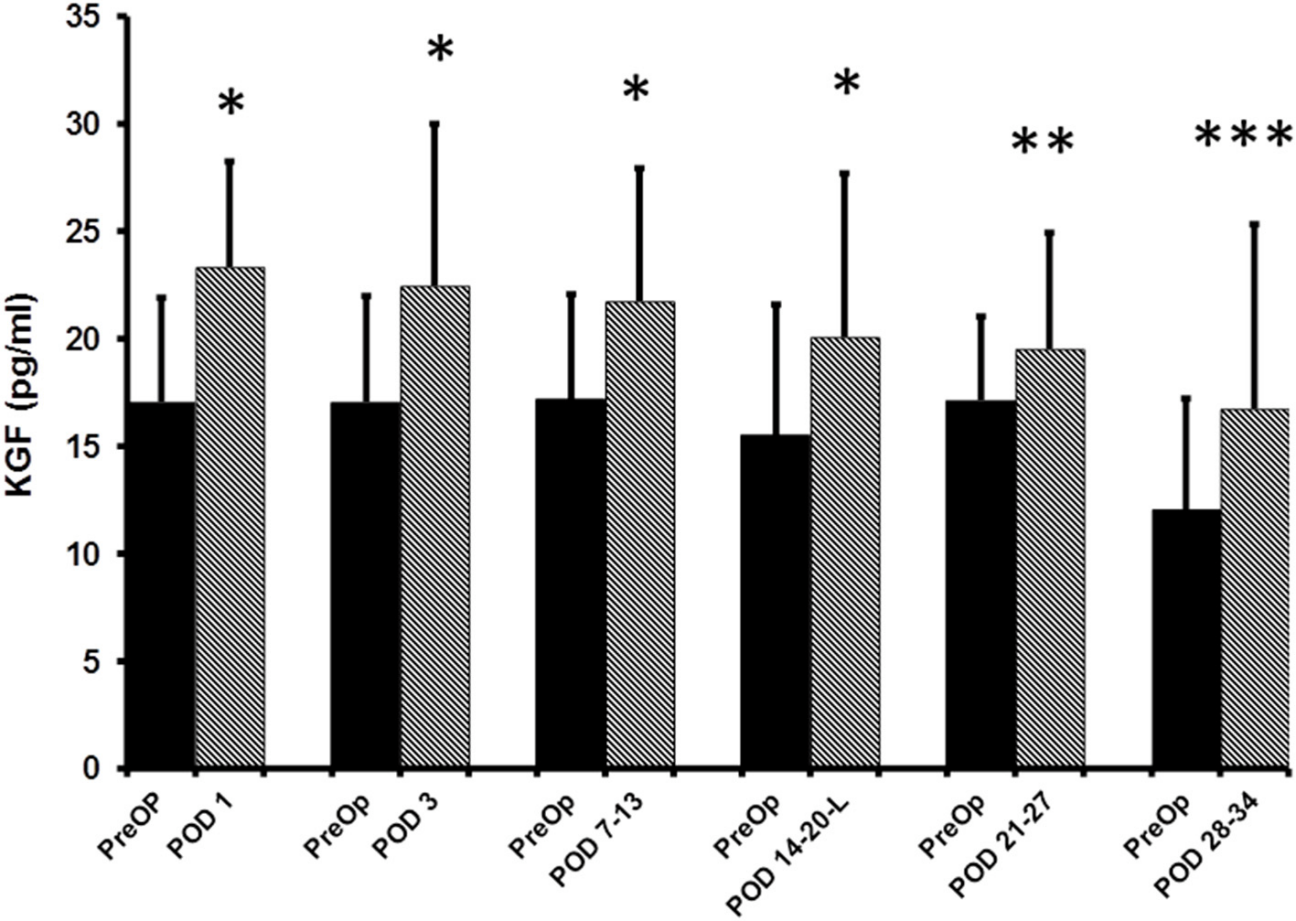
ELISA determined pre-operative (Pre-op) and post-operative plasma KGF levels of colorectal cancer patients. KGF levels are expressed as median and 75% quartile range. ^*^Pre-op vs. POD 1 (*n* = 80, *p* < 0.0001); ^*^Pre-op vs. POD 3 (*n* = 76, *p* < 0.0001); ^*^Pre-op vs. POD 7-13 (*n* = 50, *p* < 0.0001); ^*^Pre-op vs. POD 14–20 (*n* = 33, *p* < 0.0001); ^**^Pre-op vs. POD 21–27 (*n*-15, *p* = 0.03); ^***^POD 28–34 time point (*n* = 12, *p* = 0.001).

The median pre-op KGF level was 17.1 pg/ml (95%CI: 14.6–19.4; *n* = 80). When compared to pre-op levels, significant elevations in the median plasma KGF level (pg/ml) were identified at all 5 post-op time points: POD 1 (23.4 pg/ml; 95% CI: 21.4–25.9; *n* = 80, *p* < 0.0001), POD 3 (22.5 pg/ml; 95% CI: 20.7–25.9; *n* = 76, *p* < 0.0001), POD 7–13 (21.8 pg/ml; 95% CI: 17.7–25.4; *n* = 50, *p* < 0.0001), POD 14–20 (20.1 pg/ml;95% CI: 17.1–23.9; *n* = 33, *p* < 0.0001), POD 21–27 (19.6 pg/ml; 95% CI: 15.2–24.9; *n* = 15, *p* = 0.03) and on POD 28–34 (16.7 pg/ml; 95% CI: 14.0–25.8; *n* = 12, *p* = 0.001) (Figure 1). The percent increase from median baseline at each time point was 36.9% at POD 1, 31.8%; at POD 3, 26.8%; at POD 7–13, 29.7%; at POD 14–20, 14.1%; at POD 21–27, and 38.7% at POD 28–34. Of note, because the *n*'s for 5 of the 6 post-op time points vary, the mean pre-op value at these time points differs. Thus, as regards the figure displaying the results, there are two bars shown for each time point (left bar depicts the pre-op result and the right the post-op result).

No significant differences in post-operative KGF levels were noted in relation to sex, incision length, or the surgical method. Similarly, post-op KGF levels did not correlate with cancer stage. Of note, when each time point's results were considered independently, age directly correlated with KGF levels pre-operatively and at 3 of the 6 post-op time points. Also, the colon cancer subgroup had higher mean KGF levels at 5 of the 6 post-op time points although significance was reached only on POD 1. Finally, the operative length of surgery directly correlated with the mean KGF level at 1 of the 6 post-operative time points (POD 1).

## Discussion

This study of perioperative plasma KGF levels in the setting of MICR assessed 6 post-operative timepoints and revealed that blood levels are significantly elevated over pre-operative baseline for 5 weeks after surgery. As regards mean KGF values, the percent change from pre-op baseline ranged from 26.8 to 38.7% at 5 of the 6 time points. There was no association found between post-operative KGF levels and sex, length of incision, surgical method, or the final cancer stage. When each post-op time point's results were considered alone, KGF levels correlated directly with age pre-operatively and at 3 of 6 post-op timepoints. Also, the colon cancer subgroup, significantly older than the rectal cancer cohort, had significantly higher mean post-op KGF levels than the rectal cancer group at 1 of 6 time points (POD 1). Finally, the length of surgery directly correlated with the mean KGF level at 1 of the 6 post-operative timepoints. Thus, age, cancer location, and length of surgery may influence blood KGF levels.

The majority of plasma protein changes after surgery persist hours or days (12–25), however, in the last decade, increases in the blood levels of at least 12 proangiogenic proteins have been noted to persist for 3–5 weeks after resection of colorectal cancer (CRC). Included on this list are vascular endothelial growth factor (VEGF), angiopoeitin-2 (Ang-2), placental growth factor (PIGF), soluble vascular adhesion molecule-1 (sVCAM-1), Angiopoietin like 4 (ANPTL4), monocyte chemotactic protein-1 (MCP-1), human chitinase 3-like 1 (Chi3L1), matrix metalloproteinase-2(MMP-2),matrix metalloproteinase-3 (MMP-3), matrix metalloproteinase-3 (MMP-7), Chemokine (C-X-C motif) ligand 16 (CXCL 16), and Interleukin 8 (IL-8).

VEGF is a key promoter of several early steps in angiogenesis. Specifically, VEGF stimulates endothelial cell (EC) proliferation, microtubule formation, migration, and invasion supporting microvasculature development whereas Ang-2 destabilizes the connections between endothelium and perivascular cells, which enhance VEGFs effects (15). While stimulating EC proliferation and survival, PlGF recruits smooth muscle precursors that envelop developing vessels in tumors and together with VEGF produces more stable and mature vessels (17). It has been shown that the binding of sVCAM-1 to EC bound VLA4, *in vitro*, induces EC chemotaxis *via* the p38 mitogen-activated protein (MAP) kinase and focal adhesion kinase (FAK) signaling pathway which are important early step in the complex process of angiogenesis (18, 49, 50). MCP-1 is thought to facilitate angiogenesis *via* recruitment of proangiogenic protein producing monocytes and macrophages and endothelial cells into wounds and tumors (19). Macrophages in tumor stroma have been shown to express CHi3L1 which is likely to stimulate tumor angiogenesis (20, 51). MMP-2 ECM degradation releases VEGF and transforming growth factor β (TGF-β) and transformation of TGF-β into its active form is further supported by MMP-7 (25, 52–54). MMP-2, MMP-3 and MMP-7, as a proteases and regulator of cell matrix interactions, have been proposed to play a role in angiogenesis by paving the way for budding vessels and migrating cells (22, 55). CXCL16 is express on leukocytes, endothelial cells, and other tissues and its receptor CXCL6 found on cells at sites of inflammation (24). Endothelial precursor cell recruitment and angiogenesis induced by pro-inflammatory stimuli are thought to be associated with transmembrane chemokine CXCL16 and CXCR6 pair activity (56–58). IL-8 plays a role in angiogenesis as well as keratinocyte chemotaxis especially at the healing surgical wounds tissues (21). *In vitro* studies utilizing endothelial cells suggest the net effect of plasma compositional changes during 2nd and 3rd week post-operative period is proangiogenic. KGF joins the ranks of these 12 proteins whose blood levels have been shown to be elevated after MICR. As noted, among numerous other effects, these proteins all play some role in angiogenesis; KGF shares that characteristics.

Niu et al. has noted in *in vitro* studies of pancreatic ductal cells, that the binding of KGF to its receptor induces NF Kappa B activation and leads to downstream activation of a cascade of target genes that, in turn, lead to the production and release of Matrix Metalloproteinase 9 (MMP-9), Vascular Endothelial Growth Factor (VEGF) and Urokinase-type Plasminogen Activator (u-PA). These factors then induce EC proliferation and migration which leads to the branching of micro vessels and neovascularization; MMP-9 and u-PA also facilitate cell migration and invasion (32). In several studies, KGF was shown to induce neovascularization in the rat cornea, and in an *in vitro* culture study of EC's obtained from small vessels it was demonstrated that KGF induced chemotaxis, stimulated EC proliferation, activated MAPK, and helped maintain the barrier function of EC's (38, 59). These proangiogenic effects are thought to promote both wound and tumor neovascularization (48). As mentioned, KGF has also been shown to promote tumor growth.

There is considerable evidence that KGF, in the setting of KGFR expressing tumors, promotes cancer development in multiple ways including stimulation of tumor cell proliferation, motility and/or invasion (27, 30, 32, 38, 40, 60). As mentioned, numerous epithelial cancers have been shown to express KGFR including colorectal, pancreatic, gastric, and ovarian cancer (40, 42, 44, 46, 48, 60–63). The source of the KGF, in most cancers, is the peri-tumor stroma although some tumors express this protein. Numerous mechanisms have been proposed to account for KGF's tumor promoting effects. In several colon cancer studies, KGF was shown to facilitate tumor cell proliferation *via* cyclin D, an essential regulator of cell cycle progression (64, 65). Having discussed KGF's potential impact on cancers, what is the etiology of these changes after surgery?

As mentioned, the cytokines IL-1 and 6 are known to induce KGF secretion, therefore, the elevation noted during the first few days following surgery may be a consequence of the acute inflammatory response. Beyond that point, since KGF plays a role in re-epithelialization and wound angiogenesis, the healing surgical wounds may be the source of the additional protein in the circulation. KGF may follow the concentration gradient from the wound (high KGF levels) to the circulation (low KGF levels). This is in keeping with many of the other proteins, mentioned above, whose plasma levels have been shown to be persistently increased after colorectal resection (11, 15, 16, 18, 19, 21–26). In a study that simultaneously assessed plasma and wound fluid levels of 8 of these “long duration” proteins after colorectal resection, it was demonstrated that the mean wound levels of the proteins were 3 to 40 times higher than their corresponding plasma concentrations which, in turn, were significantly elevated from their pre-operative baseline blood levels (27). As noted, KGF, in addition to having proangiogenic effects, also plays a role in the wound healing process.

Although low levels of KGF are found in normal epithelium, studies have shown that notably higher levels of KGF mRNA and protein are noted along the advancing edge of wounds during re-epithelialization (66). KGF, generated by fibroblasts and other mesenchymal cells in the wound stroma, impacts KGFR expressing keratinocytes and a wide variety of epithelial cells (39, 67, 68). As noted, KGF promotes epithelial cell proliferation, differentiation, migration and has also been shown to promote wound contraction in a rodent model of diabetic wounds (27, 28). What are the potential clinical implications of the post-operative KGF changes?

In theory, a month long period of elevated plasma KGF levels might stimulate the growth of residual cancer deposits after resection of the primary tumor. This idea seems more reasonable when considered in light of the other 12 proteins whose blood levels are similarly elevated for 3–5 weeks. As regards neovascularization, the collective effect of the surgery-related plasma compositional changes is to stimulate EC proliferation, mobility, and invasion when compared to pre-operative plasma (15, 26). Thus, tumor angiogenesis may be stimulated post-operatively. The authors also believe that the long duration plasma protein changes, including the KGF elevations, may promote tumor development by directly stimulating tumor cell mitosis, mobility, and growth *via* multiple mechanisms including the inhibition of apoptosis (69–71). All of the proteins that are persistently elevated after CRC have been shown to promote tumor growth in numerous ways in addition to having proangiogenic effects. When considered in light of existing concerns regarding the possible tumor promoting effects of surgery, the development of anti-tumor treatments and strategies that can be employed perioperatively is logical.

Presently, for the vast majority of solid cancers for whom the primary treatment is surgical resection, the perioperative time period is not used for any anticancer treatment. Potential therapies that have been proposed for use in the perioperative period in either the murine or clinical setting include immunomodulation (FLT-3, GMCSF,), tumor vaccines, monoclonal antibodies to EGFR, H-2 blockers (shown to inhibit some regulatory T cells), and anti-oxidants with anti-tumor effects such as EGCG (green tea component) and siliphos (milk thistle component) (10, 11, 72–74). Clearly, more research in this area is warranted.

**Figure 2:**
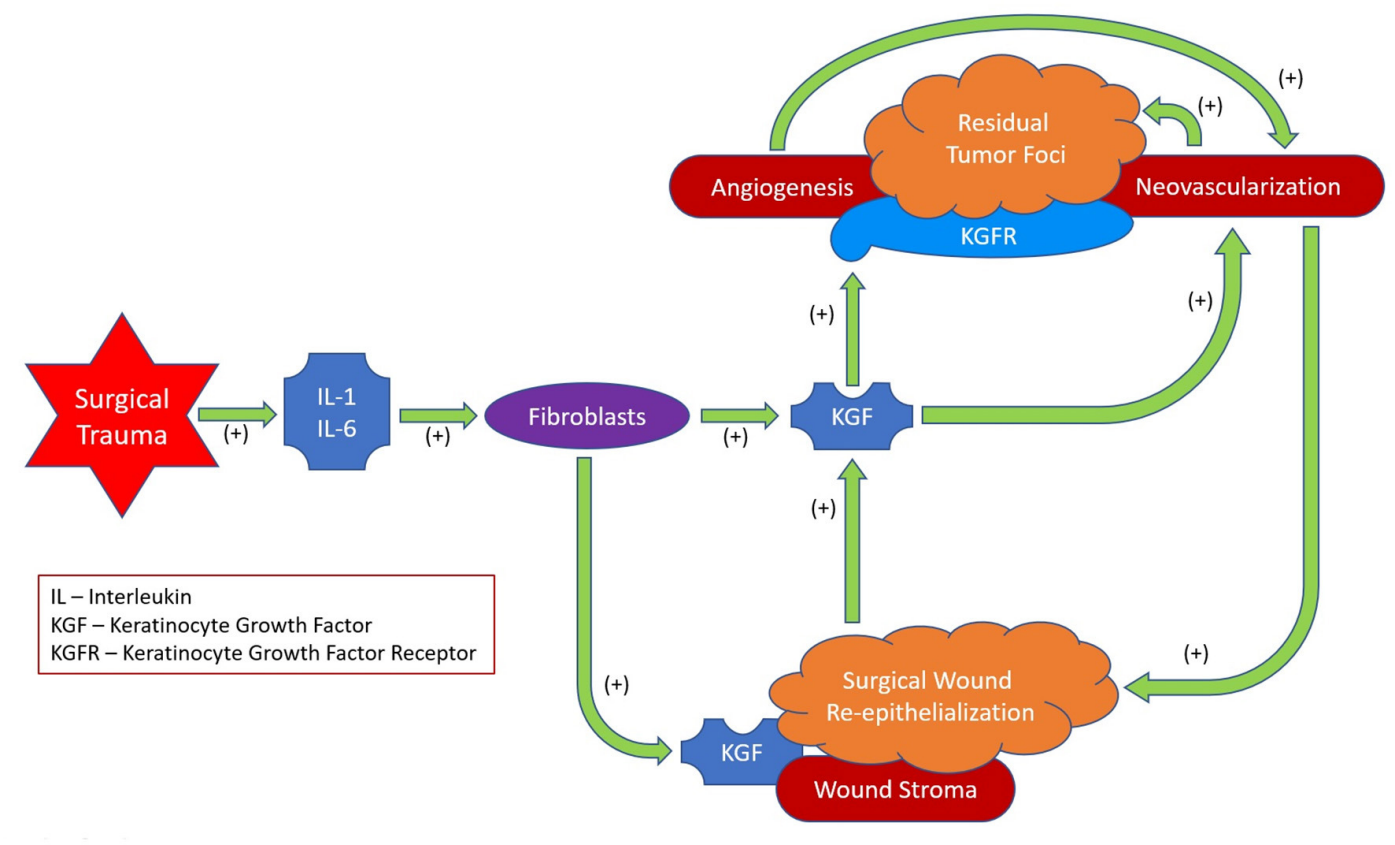
Proposed ramifications of surgical trauma related plasma KGF effects.

In conclusion, after MICR for CRC, plasma KGF levels are significantly elevated over baseline levels for 5 weeks. The etiology of these post-operative changes was not assessed in this study. However the cytokines IL-1 and IL6 are known to induce KGF secretion, therefore, the elevation noted during the first few days following surgery may be a consequence of the acute inflammatory response. Beyond that point, since KGF plays a role in re-epithelialization and wound angiogenesis, the healing surgical wounds may be the source of the additional protein in the circulation (75–78). KGF may follow the concentration gradient from the wound (high KGF levels) to the circulation (low KGF levels). The clinical importance of these findings, if any, is unclear. Because KGF's purported effects include promotion of cell turnover, mobility, and angiogenesis, during the first post-op month residual KGFR expressing tumor deposits may be stimulated to grow (Figure 2).

Weaknesses of this study include a diminishing “*n*” and the need to “bundle” specimens for the final 4 post-hospital discharge time points. In defense, outpatient weekly blood draws are not feasible and it is not possible to coordinate late sampling so that it occurs on set days. Another shortcoming is that this study includes perioperative plasma data only. Ideally, tumor expression levels of KGFR and KGF would be determined as well. A correlative tumor expression study was not conducted due to the fact that we did not have an adequate number of tumor and normal tissue samples for the patients in this study. Also, in addition to assessing CRC patients who had minimally invasive surgery, ideally, patients undergoing “open” (large incision) surgery would also have been studied. Unfortunately, our bank has very few open surgery plasma samples because the great majority of cases are done using MIS methods. In addition, the study is too small to definitively determine the role that age, tumor location, tumor stage and length of surgery have on post-op KGF levels. Further studies with a larger study group would better answer these questions.

## Summary

After MICR for CRC, plasma KGF levels are significantly elevated over baseline levels for 5 weeks. The percent change from baseline was >25% for 5 of the 6 post-op time points. No correlation between KGF levels and tumor stage, surgical technique or sex was found, however, age >60 was associated with higher levels at some time points. KGF joins a group of 12 other proteins whose levels are persistently increased after MICR. The clinical import of these findings, if any, is unclear. Because KGF's purported effects include promotion of cell turnover, mobility, and angiogenesis, during the first post-op month residual KGFR expressing tumor deposits may be stimulated to grow. Further studies of the ramifications of surgery as regards plasma protein composition are warranted as is a search for anti-cancer agents suitable for use in the perioperative period.

## Data Availability Statement

The datasets presented in this article are not readily available because all data use for the research are included in the article. Other patients information data cannot be provided as per the IRB regulations. Requests to access the datasets should be directed to rwhelan1@northwell.edu.

## Ethics Statement

The present study was conducted using material collected from patients who have consented to pre-operatively to participate in the Mount Sinai West Colorectal service's IRB approved general tissue and data banking protocol (Institutional Review Board of the Mount Sinai School of Medicine, New York NY; IRB Reference NO: GCO#1: 16-2619). The patients/participants provided their written informed consent to participate in this study.

## Author Contributions

HMCSK contributed to the conception, design, sample processing, statistical analysis, interpretation of data, and revision of the articles. HM and XY contributed to collection of human material clinical data and revision of the article. VC contributed to human sample collection, processing, analysis, and interpretation of data. AS and YH contributed to manuscript writing, interpretation of data, and revision of the articles. RW contributed to the conception, design, interpretation of data, and critical revision of the article. All authors drafted the article, made critical revisions, and approved the submitted final version of the article to be published.

## Funding

This study was supported by a generous donation from the Wade Thompson Foundation to the Division of Colon and Rectal Surgery, Department of Surgery, Mount Sinai West Hospital (Grant account no: SL55002012), New York.

## Conflict of Interest

The authors declare that the research was conducted in the absence of any commercial or financial relationships that could be construed as a potential conflict of interest.

## Publisher's Note

All claims expressed in this article are solely those of the authors and do not necessarily represent those of their affiliated organizations, or those of the publisher, the editors and the reviewers. Any product that may be evaluated in this article, or claim that may be made by its manufacturer, is not guaranteed or endorsed by the publisher.
